# Myostatin in Obesity: A Molecular Link Between Metabolic Dysfunction and Musculotendinous Remodeling

**DOI:** 10.3390/ijms27020967

**Published:** 2026-01-18

**Authors:** Leonardo Cesanelli, Petras Minderis, Andrej Fokin, Aivaras Ratkevicius, Danguole Satkunskiene, Hans Degens

**Affiliations:** 1 Institute of Sport Science and Innovations, Lithuanian Sports University, 44221 Kaunas, Lithuania; 2 Sports and Exercise Medicine Centre, Queen Mary University of London, London E1 4NS, UK; 3 Department of Health Promotion and Rehabilitation, Lithuanian Sports University, 44221 Kaunas, Lithuania; 4 Department of Life Sciences, Manchester Metropolitan University, Manchester M1 5GD, UK

**Keywords:** myostatin, obesity, ECM, skeletal muscle, tendon, mechanotransduction, fibrosis, SMAD signaling

## Abstract

Obesity is increasingly recognized not only as a metabolic disorder but also as a condition marked by the structural and functional deterioration of skeletal muscle and tendon tissues. Central to this process is the dysregulation of the extracellular matrix (ECM) resulting in fibrosis and ectopic fat accumulation, factors that contribute to impaired tissue mechanics. Myostatin (GDF-8), a member of the TGF-β superfamily, is known as a negative regulator of muscle mass. It can also mediate interaction between adipose and other tissues including muscles and tendons. In obesity, elevated myostatin levels have been reported to be associated with insulin resistance, muscle atrophy, and activation of SMAD2/3 signaling, while experimental and preclinical studies indicate that myostatin inhibition can improve glucose homeostasis and increase lean mass. Emerging evidence suggests that myostatin also plays a critical role in muscle ECM and tendon remodeling. Restoring its physiological levels may help reverse ECM disorganization and reduce tissue fragility associated with musculotendinous dysfunction. This review highlights the multifaceted role of myostatin in obesity, beyond its role in muscle catabolism, to include modulation of structural integrity, metabolism, and mechanical adaptability of the musculotendinous system. Understanding how myostatin responds to metabolic stress and affects biomechanical remodeling offers novel insights into obesity-related muscle and tendon dysfunction.

## 1. Introduction

Obesity is increasingly recognized as not only a metabolic disorder but also a condition that induces profound structural remodeling of the musculotendinous system [1,2]. This remodeling is often characterized by the accumulation of adipose tissue and fibrosis within skeletal muscle and tendons [3,4,5,6]. Central to this maladaptive tissue remodeling is the extracellular matrix (ECM), whose homeostasis in muscle and tendon is tightly regulated and highly sensitive to both metabolic and mechanical stress [7,8,9]. In obesity, this balance is frequently disrupted, leading to excessive ECM deposition and increased collagen crosslinking that ultimately affects the overall tissue mechanical behavior [3,4,5,10,11].

Among the key regulators of ECM remodeling in skeletal tissues is the transforming growth factor beta (TGF-β) superfamily, a large group of cytokines that governs cell differentiation, matrix production, and inflammatory signaling [12,13,14]. In both muscles and tendons, the TGF-β–SMAD pathway is considered a central signaling axis for collagen synthesis and regulation of fibroblast activity [13,14,15]. Elevated TGF-β activity has been consistently observed in obesity and type 2 diabetes, and is closely associated with the pathological accumulation of fibrotic tissue in various organs, including skeletal muscle [3,16,17,18,19].

A muscle-specific member of the TGF-β superfamily, myostatin (GDF-8), has emerged as a potentially significant, but still underexplored, regulator linking obesity and obesity-associated metabolic dysfunction with coordinated remodeling of skeletal muscle and tendon tissues [20,21]. Myostatin exerts its effects primarily through binding to the activin receptor type IIB (ActRIIB), in complex with type I receptors ALK4 or ALK5, leading to SMAD2/3 phosphorylation and transcriptional regulation of target genes, as demonstrated in cellular and in vivo models [22,23,24,25]. Since its discovery in 1997, myostatin has been shown to inhibit skeletal muscle hypertrophy by suppressing satellite cell activation, protein synthesis, and myogenic differentiation [26]. Mice with genetic inactivation of myostatin develop extreme muscle hypertrophy, and similar mutations in cattle, dogs, and humans confirm its critical role in regulating muscle mass [22]. Elevated circulating and local myostatin levels have been reported in both rodent models and humans with obesity, often correlating with low muscle mass, impaired insulin sensitivity, and markers of systemic inflammation [21,27,28,29]. These elevations have been linked to increased SMAD2/3 signaling and downregulation of anabolic and mitochondrial genes, suggesting a broader suppressive effect on muscle mass and metabolic function [20]. A dysfunction of brown adipose tissue (BAT) is coupled with increased production of myostatin which then mediates negative effects on muscle and other tissue health and function [30]. Furthermore, recent preclinical work shows that combining blockade of growth differentiation factor-8 (GDF8, myostatin) and activin A with GLP-1 receptor agonist therapy enhancing fat loss in diet-induced obese mice and non-human primates while preserving lean mass. This highlights the promise of dual targeting of ActRII ligands in obesity treatment strategies [31].

**Figure 1:**
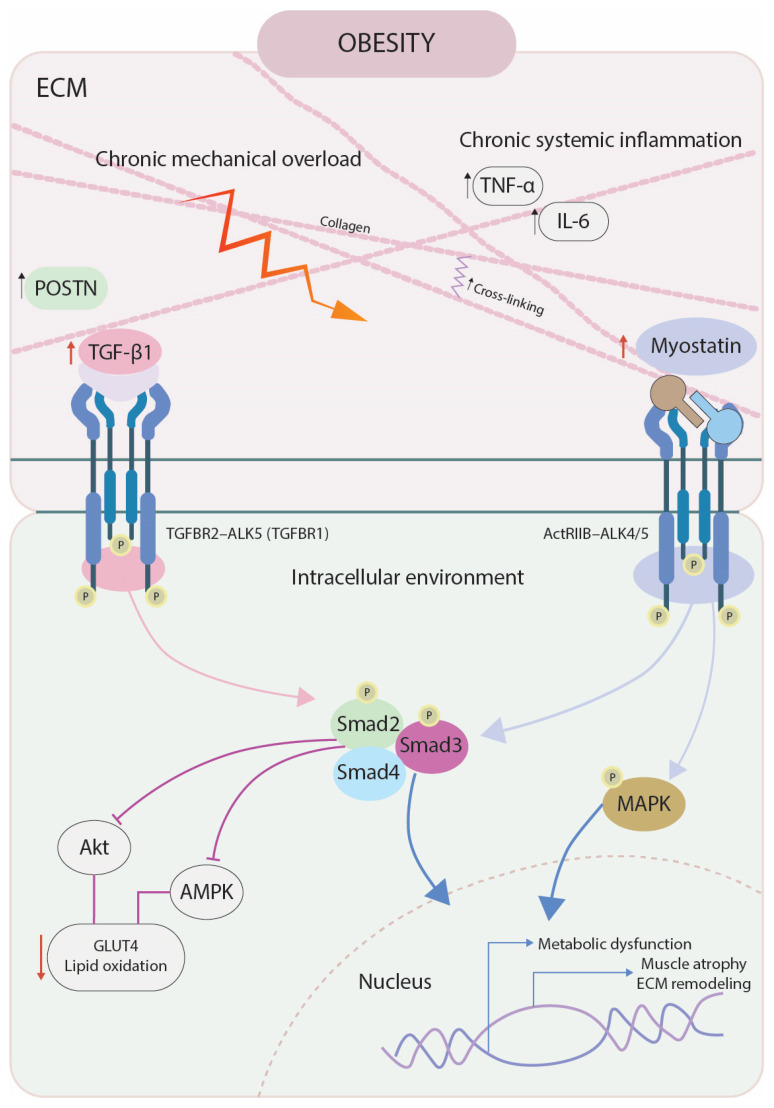
Conceptual model illustrating how obesity-associated chronic low-grade inflammation and myostatin signaling may contribute to metabolic dysfunction and extracellular matrix (ECM) remodeling in musculotendinous tissue. In obesity, elevated inflammatory mediators (e.g., IL-6) and increased myostatin levels activate ActRIIB–ALK4/5 and TGF-β receptor complexes, leading to SMAD2/3 signaling and nuclear transcriptional responses. Alongside other ECM-regulating factors such as periostin (POSTN) —a matricellular protein involved in collagen organization and fibrotic signaling [45]—these pathways are associated with altered collagen organization, increased ECM turnover, and adipose tissue infiltration. Downstream inhibition of Akt/AMPK signaling contributes to impaired GLUT4 translocation and lipid oxidation, linking structural remodeling with metabolic dysfunction.

While myostatin has been extensively studied for its canonical role as a negative regulator of muscle growth, accumulating evidence suggests that it also influences ECM turnover and the fibro-adipogenic response affecting tissue mechanical properties in muscle and tendon, particularly under metabolic challenge [32,33,34,35,36,37,38], and especially in the context of obesity [20,36,37]. Myostatin may stimulate fibroblasts and ECM-producing cells, increasing collagen deposition, altering structural features and increasing the stiffness in musculotendinous tissues [33,36,37,39,40]. In vitro studies demonstrate that myostatin stimulates fibrotic gene expression and impairs ECM degradation by modulating matrix metalloproteinase activity [41]. These observations raise important questions about myostatin’s contribution to the musculotendinous remodeling commonly seen in obesity, where increased ECM remodeling and deposition is a major determinant of increased muscle-tendon fragility, dysfunction, and exercise intolerance (Figure 1) [3,4,10,42].

While several studies have examined the metabolic effects of myostatin inhibition—including enhanced glucose uptake, increased insulin sensitivity, and resistance to diet-induced obesity—fewer have investigated how myostatin signaling interacts with mechanical loading, ECM remodeling, and tendon adaptation under metabolic stress [20,43]. This is particularly relevant for individuals with obesity, where increased body mass imposes chronic overload on musculotendinous structures, yet often results in impaired adaptations to physical activity [32,44]. It is plausible that elevated myostatin levels in obesity may suppress not only muscle growth but also influence mechanotransduction pathways that play a role in ECM reorganization, thereby contributing to both metabolic inflexibility and mechanical dysfunction [3,20,32]. Accordingly, the aim of this brief narrative review is to critically examine current evidence supporting myostatin as a molecular regulator linking metabolic dysfunction in obesity with coordinated remodeling of muscle and tendon tissues, while highlighting areas where mechanistic data remain limited.

## 2. Materials and Methods

The literature search was conducted between May 2025 and July 2025 using four major electronic databases: PubMed, Scopus, Web of Science, and Embase (Elsevier). The search strategy was designed to identify studies addressing the relationship between myostatin and obesity, with particular attention to metabolic dysfunction and musculotendinous remodeling. The following core search terms were used in various combinations: myostatin (including “growth differentiation factor 8” and GDF8), obesity (including “high-fat diet” and “diet-induced obesity”), skeletal muscle and tendon, and extracellular matrix remodeling (including fibrosis and collagen-related terms). In PubMed, field tags (e.g., [Title/Abstract]) were applied where appropriate, while equivalent keyword-based strategies were adapted for Scopus, Web of Science, and Embase according to each database’s syntax and indexing structure.

Myostatin was first described in 1997, but its involvement in obesity- and high-fat diet-related conditions has primarily been explored since the early 2000s [20,26]. Therefore, the search was limited to studies published within the last 25 years.

Study selection was performed in two stages. Titles and abstracts were initially screened by one author (L.C.) to assess relevance. Full texts of potentially eligible articles were then reviewed, and final inclusion was confirmed by a second author (H.D.). Additional relevant studies were identified by screening the reference lists of included articles.

Studies were included if they investigated myostatin in the context of obesity or obesity-associated metabolic stress, including in vitro, ex vivo, in vivo, animal, and human studies. Exclusion criteria comprised case reports, studies not reporting myostatin-related outcomes, and articles not written in English. Articles that did not directly meet inclusion criteria but were cited for background or conceptual context were explicitly limited to framing or hypothesis-generating sections and were not used to support primary mechanistic conclusions.

## 3. Myostatin in Obesity: Systemic Metabolic Roles

Evidence across human and animal models indicates that obesity is frequently, but not always, associated with increased myostatin expression at both systemic and tissue levels, with reported effects on muscle plasticity, glucose metabolism, and inflammatory status. Importantly, findings vary depending on the biological compartment assessed (e.g., serum/plasma versus tissue), the molecular readout (mRNA versus protein), and cohort characteristics such as age, insulin sensitivity, and inflammatory burden. Myostatin mRNA levels are increased in both adipose and skeletal muscle tissue from genetically obese, leptin-deficient *ob*/*ob* mice and from wild-type mice fed a high-fat diet for 1 month [21]. Proteomic analyses of conditioned media from myotubes derived from extremely obese women have demonstrated increased myostatin secretion in vitro, while in vivo plasma myostatin concentrations correlated with insulin resistance in the same cohort [27]. Subsequent genetic studies have reported associations between polymorphisms in the myostatin gene (e.g., A55T, K153R, rs1805086) and greater adiposity, lower lean mass, and increased susceptibility to obesity across different populations [46,47,48]. These associations were independent of classical metabolic markers, suggesting that variation in myostatin signaling may contribute to interindividual differences in body composition, although causal relationships cannot be inferred from these data alone.

Animal models have helped clarify causal mechanisms. Myostatin depletion has been shown to limit weight gain and improve glucose homeostasis during prolonged high-fat feeding [49], while obesity- and exercise-associated changes in myostatin and ActRIIB expression suggest a link between myostatin signaling and metabolic regulation in insulin-resistant states [50]. Two weeks of daily leptin injections resulted in a reduction of myostatin mRNA levels in both muscle and adipose tissue in adult *ob*/*ob* mice [21], while post-developmental depletion of myostatin improved glucose clearance, it did not entirely prevent fat accumulation during prolonged overfeeding [49]. Collectively, myostatin seems to respond to energy homeostasis regulation, being elevated in response to high-fat diet [50], and dropping following weight or adipose tissue loss [21], supporting its role as a modulator rather than a unidirectional driver of metabolic dysfunction.

Similarly, in humans high levels of myostatin are associated with metabolically unhealthy obesity, hyperinsulinemia, and increased TNF-α and CRP levels [51,52,53], as well as sarcopenia in older individuals [54]. Despite consistent findings of elevated myostatin in obesity, some variability remains. For instance, depending on insulin sensitivity, age, or inflammation status studies in some cases reported unchanged myostatin expression in individuals with obesity [55,56]. Weight loss, on the other hand, has been reported to induce a reduction of myostatin mRNA levels in muscle biopsies from obese patients that appears to predict therapeutic responsiveness [57,58]. Gradual and combined exercise-diet approaches reduce myostatin more effectively than rapid weight loss alone, and helped to preserve lean mass and improve metabolic flexibility [59,60]. However, only individuals with initially elevated myostatin levels showed significant glucose metabolism improvements after hypocaloric dieting, suggesting baseline myostatin as a potential stratification tool for dietary planning [61].

The significance of myostatin as an effector is demonstrated by studies showing that experimental manipulation of myostatin expression can dramatically affect the development of obesity in mice. Muscle-specific overexpression of myostatin caused a decrease in muscle mass and an increase in epididymal fat pad mass [28]. In contrast, crossing myostatin-null mice with genetically obese *ob*/*ob* or agouti yellow mice attenuated the excessive adipose tissue accumulation, hyperglycemia, hyperlipidemia, and hyperinsulinemia typically observed in these genetically obese mice models [62]. Postnatal transgenic overexpression of the inhibitory prodomain of myostatin in mice also increased muscle growth and attenuated the effects of 2-month high-fat diet on adipose tissue expansion as well as circulating insulin and glucose levels [63,64]. Injection with a soluble ActRIIb receptor—a non-selective ligand trap that disrupts signaling of myostatin as well as related TGF-β family ligands such as activins—increased muscle mass and decreased fat mass in mice fed either a high-fat or standard chow diet [65].

Although obesity-induced increases in myostatin and ActRIIB receptor expression have been observed in adipose tissue, probably contributing to enhanced adipogenic differentiation in vitro [21,25,66,67,68,69], myostatin expression levels in adipose depots remain substantially lower than those detected in skeletal muscle [21]. Functional evidence strongly supports this tissue hierarchy: in mice, selective inhibition of myostatin signaling in skeletal muscle, but not in adipose tissue, led to substantial protection against high-fat diet–induced metabolic deterioration. Specifically, muscle-specific inhibition preserved lean mass, reduced adiposity and adipocyte size, and improved glucose tolerance, insulin sensitivity, and lipid profiles. In contrast, adipose-specific inhibition failed to induce comparable effects [70]. Similarly, administration of a myostatin neutralizing antibody in *ob*/*ob* mice reduced circulating glucose and fatty acid levels, and increased energy expenditure and activity without altering fat mass [71].

In sum, these findings identify skeletal muscle as the principal tissue through which myostatin contributes to obesity-related metabolic dysfunction. While reduced muscle mass may impair glucose uptake and metabolic flexibility, emerging evidence suggests that myostatin may also influence ECM remodeling and fibro-adipogenic infiltration—processes that are well established in obesity but for which myostatin-specific causal data remain limited [3,72,73,74]. As a member of the TGF-β superfamily, myostatin can activate downstream fibrotic signaling pathways, supporting a plausible mechanistic link between metabolic stress and structural degeneration of the musculotendinous system (Figure 1 and Table 1).

## 4. Myostatin as a Mediator of Obesity-Induced Musculotendinous Remodeling

Elevated myostatin levels in obesity appear to contribute not only to muscle atrophy but also to broader complications of musculotendinous structure and function. Myostatin upregulation in skeletal muscle of individuals with obesity, observed in both humans and animal models, correlates with impaired muscle regeneration, increased SMAD signaling, and downregulation of myogenic regulatory factors such as MyoD and myogenin, thereby disrupting myogenesis and exacerbating muscle weakness [27,55]. Chronic obesity-associated conditions, including insulin resistance and inflammation, amplify these effects, creating a “maladaptive” muscle environment characterized by fibrosis, altered contractile properties, and reduced mitochondrial function [75,76]. Supporting this, Rasool et al. (2018) reported that a high-fat, high-sucrose diet consistently leads to obesity-induced myodegeneration, muscle atrophy and strength loss, with myostatin likely playing a contributing role [77]. Similarly, chronic overnutrition in animal models increases myostatin expression and contributes to long-term deficits in muscle development and load-bearing capacity [78,79].

In addition to its well-established role as a negative regulator of muscle mass, myostatin also influences tendon structure and mechanical properties. In mice lacking myostatin, tendons are smaller, more brittle, and hypocellular, with reduced fibroblast density and decreased expression of type I collagen, as well as downregulation of scleraxis, and tenomodulin—key regulators of tendon fibroblast proliferation and ECM synthesis [39]. These structural alterations are accompanied by increased tendon stiffness, greater peak stress, and lower peak strain [39]. In a separate study, myostatin-deficient muscles exhibited a greater reduction in stiffness following mechanical damage, suggesting that myostatin dysfunction compromises muscle structural integrity [33]. Studies in tendon fibroblasts showed that myostatin may mediate this effect via activation of p38 MAPK and Smad2/3 signaling that was associated with cell proliferation and matrix production [39]. Furthermore, myostatin has been associated with muscle fibroblasts proliferation and regulation and the production of ECM proteins both in vitro and in vivo, involving the activation of Smad, p38 MAPK and Akt pathways [36,37]. These findings support a regulatory role for myostatin in fibroblast behavior but do not, on their own, establish in vivo tendon remodeling during obesity.

Serum myostatin levels have been reported to correlate positively with hyperinsulinemia, independent of muscle mass in obese patients [80]. Interestingly, muscle-targeted inhibition of myostatin improved insulin sensitivity, reduced fat mass and increased lean mass, supporting a muscle-centric mechanism underlying the metabolic benefits of myostatin [70]. The significance of myostatin is reflected by the dual blockade of GDF8 and activin A during GLP-1 receptor agonist-induced weight loss that not only resulted in greater fat loss but also prevented muscle wasting and improved metabolic measures in both obese mice and non-human primates, suggesting that simultaneous inhibition of multiple negative regulators of muscle mass optimizes body composition outcomes of anti-obesity therapies [31].

The association between elevated myostatin and intramuscular fat accumulation [54], frequently observed in obesity [3,5,6], may be explained by its regulatory role in both metabolic and structural signaling pathways within skeletal muscle. In vivo studies using myostatin-deficient mice demonstrate reduced whole body white and brown adipose tissue mass despite accelerated adipogenic differentiation and expansion of adipose progenitor pools, suggesting that decreased fat mass results primarily from nutrient repartitioning toward skeletal muscle rather than suppression of adipocyte formation [81]. These findings highlight a complex role for myostatin in adipose tissue biology, reconciling reduced adiposity with preserved or even enhanced adipogenic capacity.

Clinically, Ishibashi et al. (2024) reported lower circulating myostatin levels in individuals with sarcopenic obesity compared with non-sarcopenic obese controls, identifying serum myostatin as a potential early biomarker of muscle wasting in obesity [82]. Further supporting this view, Yan et al. (2021) contrasted pathological obesity induced by a high-fat diet in mice with metabolically healthy obesity in pre-hibernating ground squirrels [83]. Pathological obesity was characterized by reduced muscle mass, suppressed Akt/mTOR signaling, increased protein degradation via the ubiquitin–proteasome and calpain pathways, and elevated myostatin expression, changes consistent with catabolic muscle remodeling [83]. In contrast, the pre-hibernation model maintained physiological myostatin levels, preserved muscle integrity, and supported regenerative signaling [83]. Thus, this reinforces myostatin’s role as a mediator of maladaptive tissue responses.

Upon binding to the ActRIIB receptor, myostatin activates SMAD2/3 signaling, which suppresses the IGF-1–Akt–mTOR axis and inhibits muscle protein synthesis. In experimental cell and animal models, myostatin signaling has also been reported to impair insulin sensitivity through reductions in AMPK activity and GLUT4 translocation to the muscle plasma membrane [43]. Beyond these canonical effects, SMAD signaling—particularly when phosphorylated at its linker region by MAPKs—also governs ECM remodeling by promoting the transcription of tissue fibrosis and collagen turnover related genes [36,84,85]. Indeed, in rodent models of metabolic overload and hyperglycemia, chronic activation of myostatin–SMAD2/3 pathway has been associated with increased collagen deposition and fibrosis, potentially extending to musculotendinous junctions and contributing to impaired force transmission [51,75]. It has been proposed that such fibrotic remodeling may extend to musculotendinous junctions and contribute to impaired force transmission, although direct evidence for MTJ-specific ECM remodeling in obesity remains limited.

Elashry et al. (2019) further demonstrated that myostatin-null mice fed a high-fat diet undergo muscle-specific remodeling, including fiber type shifts and changes in fiber size, suggesting that myostatin’s role in adaptations to obesogenic stress is largely muscle-specific [86]. Thus, chronic myostatin overexpression in obesity may not only impair metabolic function but also promote ECM remodeling toward fibrosis, potentially leading to increased stiffness and reduced mechanical resilience [33,36,39].

Together, these findings support the hypothesis that elevated systemic and local myostatin expression contributes not only to metabolic dysfunction but also to maladaptive remodeling of muscle and tendon tissues during obesity (Table 1). Therapeutically targeting the myostatin axis may therefore represent a promising strategy to preserve muscle mass and potentially protect musculotendinous structural integrity under obesogenic conditions, although tissue-specific effects and mechanisms require further investigation.

## 5. Conclusions

Myostatin lies at the interface between metabolic dysfunction and structural alterations of skeletal muscle in obesity, acting not only as a suppressor of muscle growth but also as a regulator of extracellular matrix (ECM) remodeling with consequences for tissue architecture and function. While its canonical role in muscle mass regulation through inhibition of the IGF-1/Akt–mTOR axis is well established, accumulating evidence supports broader actions mediated by SMAD2/3 signaling that extend to fibrosis, collagen turnover, and maladaptive remodeling of obese skeletal muscle.

In obesity, elevated myostatin is robustly associated with impaired insulin signaling, increased intramuscular fat infiltration, and altered muscle structure, whereas experimental suppression of myostatin signaling improves glucose homeostasis and insulin sensitivity. At the same time, emerging data indicate that myostatin modulation can influence ECM dynamics, suggesting that metabolic and structural outcomes are closely intertwined and context dependent. These observations support the concept that myostatin is not merely a downstream consequence of obesity-induced muscle atrophy, but an active contributor to metabolic regulation and muscle tissue remodeling.

To date, human evidence remains largely associative and is influenced by heterogeneity in obesity phenotypes, analytical approaches, and myostatin assessment strategies (tissue vs. circulating; mRNA vs. protein). Moreover, while tendon and musculotendinous junction involvement is supported by indirect and preclinical data, direct structural and mechanical assessments in obesity are limited. Future studies should therefore combine standardized myostatin profiling with longitudinal and interventional designs and include integrated evaluation of the muscle–tendon unit under obesogenic conditions. Such approaches are likely to be critical for refining the therapeutic potential of targeting the myostatin axis while preserving tissue integrity.

## Figures and Tables

**Table 1:** Conceptual summary of evidence linking myostatin signaling with obesity-associated metabolic and musculotendinous alterations.

Evidence Context	Obesity Phenotype/Model	Systemic Myostatin (Circulating)	Muscle/Tendon Myostatin Signaling	Metabolic Consequences	Musculotendinous/ECM Consequences	Direction of Evidence
Human	Obesity without sarcopenia	↑ or unchanged	↑ (inferred from tissue markers)	Insulin resistance; metabolic dysfunction	Increased ECM remodeling; reduced tissue adaptability	Consistent
Human	Sarcopenic obesity	↓	↓ (reflecting reduced muscle mass)	Muscle loss; impaired metabolic health	Compromised muscle–tendon integrity	Emerging
Animal	Diet-induced obesity	↑	↑	Weight gain; glucose intolerance	Increased collagen turnover; ECM remodeling	Consistent
Animal	Genetic myostatin deficiency	↓ or normal	↓	Reduced adiposity via nutrient repartitioning	Altered ECM organization; preserved muscle mass	Consistent
Animal	Muscle-targeted myostatin inhibition	↓	↓ (muscle-specific)	Improved glucose homeostasis; reduced fat mass	Limited or indirect ECM effects	Consistent
Animal	Combined anti-obesity pharmacotherapy + ActRII ligand blockade	↓	↓	Preserved lean mass; enhanced fat loss	Not yet fully characterized	Emerging

## Data Availability

No new data were created or analyzed in this study. Data sharing is not applicable.

## Abbreviations

The following abbreviations are used in this manuscript:

ActRIIB	Activin receptor type IIB
Akt	Protein kinase B
AMPK	AMP-activated protein kinase
BAT	Brown adipose tissue
CRP	C-reactive protein
ECM	Extracellular matrix
GDF-8	Growth differentiation factor 8 (myostatin)
GLUT4	Glucose transporter type 4
HFD	High-fat diet
IGF-1	Insulin-like growth factor 1
MAPK	Mitogen-activated protein kinase
mRNA	Messenger ribonucleic acid
mTOR	Mechanistic target of rapamycin
POSTN	Periostin
SMAD2/3	Mothers against decapentaplegic homologs 2 and 3
TGF-β	Transforming growth factor beta
TGF-β1	Transforming growth factor beta 1
TNF-α	Tumor necrosis factor alpha
